# Prostatic Leiomyoma: An Uncommon Diagnosis Found in Clinically Suspected Cases of Severe Benign Prostatic Hyperplasia Without Elevated Serum Total Prostate-Specific Antigen Levels

**DOI:** 10.7759/cureus.37035

**Published:** 2023-04-02

**Authors:** Jesse Qiao

**Affiliations:** 1 Pathology, Texas Tech University Health Sciences Center El Paso Paul L. Foster School of Medicine, El Paso, USA

**Keywords:** urinary obstruction, psa, stromal nodule, leiomyoma, prostate

## Abstract

Leiomyomas of the prostate are uncommon benign tumors. We report the case of a 67-year-old man who underwent an emergent, open prostatectomy for symptomatic relief of clinically severe benign prostatic hyperplasia (BPH). Ultrasound showed a severe prostatic enlargement with urinary tract obstruction. Gross pathology findings showed a 134-g prostate gland containing a 2.5-cm-long, well-circumscribed lesion. Histological findings showed a bland, smooth muscle neoplasm with positive staining for smooth muscle markers. No necrosis, nuclear atypia, or mitoses are identified. In such cases, gross and microscopic examination of adequately sampled lesions is necessary to assure a conclusive diagnosis and to exclude overt stromal malignancies, such as leiomyosarcoma.

## Introduction

Leiomyomas of the prostate are very uncommon benign mesenchymal tumors. This condition was first described by Kaufman and Berneike in 1951 [1]. Their definition required the tumor to be comprised of smooth muscle completely lacking glandular tissue [2]. Fewer than 50 cases of prostatic leiomyoma have been reported [3]. In such cases, histopathological examination of tissue from adequately sampled lesions is necessary to assure a conclusive diagnosis and to exclude overt stromal malignancies, such as leiomyosarcoma. We present a patient who was incidentally diagnosed with prostatic leiomyoma after undergoing an open prostatectomy for symptomatic relief of clinically suspected severe benign prostatic hyperplasia (BPH).

The historical case reports that described prostatic leiomyomas, including those described by Kaufman et al., were published prior to the advent of serum prostate-specific antigen (PSA) testing [1]. Since the diagnosis of prostatic leiomyoma is exceedingly rare, to date there has not been a meta-analysis of the correlation between the clinical and histopathological findings, in conjunction with laboratory findings, for cases of prostatic leiomyomas.

## Case presentation

A 67-year-old Hispanic male with no known allergies presented to the emergency department with hematuria and acute urinary retention for several hours. He rated his pain as 5/10 and described it as pressure. He had never had these symptoms before. A month before, he described having a "good urinary stream" with nocturia two times per night. The patient had a significant medical history of hypertension, obesity, and an enlarged prostate. His at-home medications were tamsulosin, losartan, metoprolol, and low-dose aspirin. According to the patient, his outpatient urologist described a "lump" on the right side of his prostate gland but stated that his PSA levels are not elevated, thus not requiring alpha-reductase inhibitors, and may be followed up on a yearly basis.

The patient’s past surgical history is significant for left total knee arthroplasty, right inguinal hernia repair, appendectomy, lumbar spine surgery, and cataract removal with the placement of intra-ocular lens implants. The patient’s weight and height were 106 kg and 167 cm, respectively, and his body mass index (BMI) was 37.6 kg/m^2^. He reported that he did not use tobacco or alcohol.

**Figure 1:**
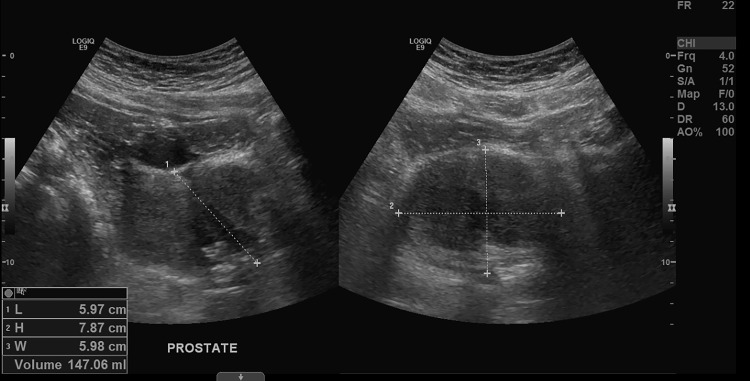
Retroperitoneal ultrasound Retroperitoneal ultrasound demonstrated marked prostatic gland enlargement (7.9 cm × 5.9 cm × 5.9 cm) with median lobe hypertrophy.

The patient was initially seen in the emergency department with a Foley catheter in place and subsequently discharged. However, the next day, the patient experienced significant hematuria with an increase in pain and returned to the emergency department. Urology was consulted for gross hematuria superimposed on urinary retention. Retroperitoneal (renal, urinary bladder, and prostate) ultrasound (Figure 1) showed marked enlargement of the prostate gland (7.9 cm × 5.9 cm × 5.9 cm, estimated at 147 mL in volume) with median lobe hypertrophy, marked distention of the urinary bladder, and no evidence of hydronephrosis in the kidneys. No mass lesions are seen in the urinary bladder or kidneys. A three-way Foley urinary catheter was inserted, which drained 700 mL of pink urine and occasional small blood clots. Initial workup showed a PSA level of 3.4 ng/mL (reference range: <4.0 ng/mL).

Given the size of the prostate gland on ultrasound (147 mL) and the hematuria suspected secondary to urinary retention, the patient was evaluated for transurethral resection of the prostate (TURP) versus a simple prostatectomy procedure. The patient elected and subsequently underwent a simple prostatectomy. He was discharged home in stable condition. Subsequent post-operative outpatient follow-up was unremarkable.

**Figure 2:**
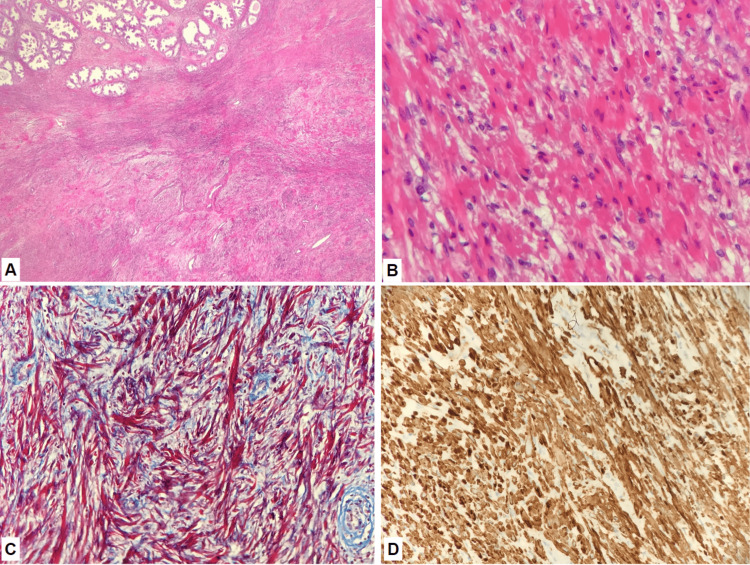
Microscopic images of the prostatic lesion (A) Low power magnification (H&E, 40×); (B) high power magnification (H&E, 400×); (C) trichrome stain demonstrating smooth muscle fibers (Trichrome, 200×); (D) desmin demonstrating positive staining (desmin, 200×).

The gross specimen weighed 134 g and consisted of a tan-white, firm, well-circumscribed lesion, 2.5 cm in greatest dimensions, adjacent to tan-yellow, rubbery-nodular prostatic tissue. Microscopic sections (Figure 2A) demonstrated a partially circumscribed proliferation of smooth muscle tissue with no intervening prostatic glands, abutting the usual areas of glandular and stromal hyperplasia. No atypical nuclei, mitoses, necrosis, or overt malignant features were identified at high power (Figure 2B). The trichrome stain (Figure 2C) highlighted red smooth muscle fibers and intervening blue fibrovascular tissue. Tumor cells were positive for smooth muscle actin and desmin (Figure 2D). Tumor cells are negative for CD34, CD117, and S-100 immunohistochemical markers. Less than 2% of Ki-67-positive cells were observed. CD99 staining was non-contributory to the final diagnosis. Given the lesion’s size, circumscription, lack of overt malignant features, and immunoprofile, the final diagnosis was a benign prostatic leiomyoma.

## Discussion

Leiomyomas can develop in any organ containing smooth muscle tissue. The pathogenesis of prostatic leiomyomas includes chronic inflammatory or infectious disease processes in Mullerian embryonic remnants, peri-glandular smooth muscle, or the prostatic capsule [3]. In the genitourinary system, this tumor has been found in the kidney, ureter, bladder, urachus, prostate, urethra, and seminal vesicles [4]. More commonly, these benign tumors appear in the gastrointestinal tract and the female genital tract [4].

Histopathological examination is essential for a definitive diagnosis of prostatic leiomyoma, and the size of the lesion (2.5 cm, well-circumscribed in our case) should prompt consideration of stromal neoplasms aside from fibromuscular nodules. Similar to its uterine counterpart, morphologic features of leiomyomas include bundles of smooth muscle fibers with elongated, tapered nuclei, increased mitoses, and no nuclear or cytological atypia or the presence of necrosis. Special (trichrome) and routine immunohistochemical staining techniques can be very helpful in confirming whether differentiated smooth muscle tissue is present in these tumors, as opposed to strong, intense blue staining of fibrous tissue in fibromuscular nodules. In general, smooth muscle actin and desmin staining in prostate tissue specimens from patients with leiomyomas are positive, and Ki-67 staining should demonstrate minimal mitotic activity, a finding consistent with the benign nature of these types of tumors.

A review of the published literature on leiomyomas identified six [3-8], single-patient case reports of elderly (age range: 54-82 years) males diagnosed with a prostatic leiomyoma [3-8]. All but one case report have PSA levels reported (Table 1).

**Table 1:** List of reported cases of prostatic leiomyoma PSA: prostate specific antigen; BPH: benign prostatic hyperplasia

Reference	Patient age (years)	Alpha blocker medication	Presentation	Clinical suspicion	Prostate size, mL or weight, g	Serum PSA (ng/mL)
Our patient	67	Yes	Acute urinary retention, gross hematuria	BPH	147 mL (134 g)	3.4
3	54	No	Enlarged prostate on rectal examination	Hypervascular tumor, STUMP on needle biopsy	Not known	0.9
4	68	Not known	Acute urinary retention	History of urinary obstruction x2 years	Not known	0.3
5	70	Yes	Scheduled prostatectomy for adenocarcinoma	Prostatic adenocarcinoma on needle biopsy	190 mL (210 g)	9.8
6	82	Yes	Complete urinary retention, gross hematuria	BPH	125 mL	1.9
7	62	Not known	Acute urinary retention, gross hematuria	BPH	103 g	3.2
8	57	No	Severe urinary obstruction	BPH	Not known	Not known

Two of the case reports mentioned the administration of alpha-blockade agents to treat BPH [5,6]. All patients presented with prostamegaly and a presumptive diagnosis of BPH, with the exception of one who had a known biopsy-proven adenocarcinoma [5]. In three reports, patients presented with acute urinary retention and two with gross hematuria [4,6,7]. In all reports, the diagnosis of prostatic leiomyoma was unexpected or incidental in relation to the clinical history. The serum PSA levels ranged from 0.9 to 3.2 ng/mL, with the exception of one patient who presented with a PSA level of 9.8 ng/mL and a known diagnosis of prostatic adenocarcinoma that would account for the elevated PSA level [5].

It appears that the clinical presentation of a prostatic leiomyoma is similar or nearly identical to cases of BPH, which is far more common and prevalent in the general population, reaching up to 80% of individuals over the age of 80 [9]. However, the serum total PSA levels were less than 4.0 ng/ml in 4 out of the 5 case reports and in our patient. While our data are limited, there may be a correlation between a prostate volume over 120 ml and a prostate weight over 100 g as considerations for a leiomyoma, particularly with the presence of single and large lesions. Not surprisingly, for all identified case reports and for our patient, the diagnosis of prostatic leiomyoma was based on histopathology findings independent of any clinical findings.

Limited data are available from which to draw conclusions on the treatment effect of alpha-blockade medications and their association with a diagnosis of leiomyoma. The emphasis of this case report and analysis rests on the uncommon presentation of clinically suspected BPH with PSA levels less than 4.0 ng/ml. Serum PSA increases with age and is frequently a good predictor of prostate volume. Patients with a mean prostatic volume of 37.9 and 64.3 mL correlate with mean serum PSA levels of 6.1 and 10.4 ng/ml, respectively [10].

TURP may present as an equally feasible treatment method when compared to an open simple prostatectomy procedure when managing large prostates [11]. While TURP procedures are associated with fewer blood transfusions and shorter hospital stays, they require longer operating times when compared to a simple prostatectomy [12]. Simple prostatectomy procedures may allow for better visualization of the prostate gland undergoing enucleation and remain an alternative option when complete removal of prostatic tissue is desired.

## Conclusions

Prostatic leiomyoma, along with other benign and malignant stromal tumors of the prostate, should be considered by clinicians when evaluating a patient with an enlarged prostate and evidence of an atypical mass in the prostate. These tumors are typically identified after transurethral resection of the prostate or prostatectomy for symptomatic relief of acute urinary retention and histopathological examination of the entire excised tumor specimen. A thorough sampling of the tumor is necessary to exclude overt cellular atypia, necrosis, and increased and atypical mitoses. On the other hand, the presence of all of these findings would strongly favor a histopathologic diagnosis of malignancy. Fortunately, patients with benign stromal tumors have a very favorable prognosis, especially when these tumors are completely resected. Management may include an open simple prostatectomy for complete removal versus transurethral resection and laser prostatectomy methods.
